# Urea Level and Depression in Patients with Chronic Kidney Disease

**DOI:** 10.3390/toxins16070326

**Published:** 2024-07-22

**Authors:** Hélène Levassort, Julie Boucquemont, Oriane Lambert, Sophie Liabeuf, Solene M. Laville, Laurent Teillet, Abdel-Hay Tabcheh, Luc Frimat, Christian Combe, Denis Fouque, Maurice Laville, Christian Jacquelinet, Catherine Helmer, Natalia Alencar de Pinho, Marion Pépin, Ziad A. Massy, on behalf of CKD-REIN Study Collaborators

**Affiliations:** 1 Geriatrics, Hôpital Ambroise-Paré, Assistance Publique des Hôpitaux de Paris (APHP), UVSQ, 9 Avenue Charles de Gaulle, F-92100 Boulogne-Billancourt, Francemarion.pepin@aphp.fr (M.P.); 2 Centre for Research in Epidemiology and Population Health (CESP), Clinical Epidemiology Team, Inserm U1018, Paris-Saclay University, 12 Avenue Paul Vaillant Couturier, F-94800 Villejuif, Franceoriane.lambert@inserm.fr (O.L.); abdel-hay.tabcheh@inserm.fr (A.-H.T.); natalia.alencar-de-pinho@inserm.fr (N.A.d.P.); 3 Pharmacoepidemiology Unit, Department of Clinical Pharmacology, Amiens-Picardie University Medical Center, F-80054 Amiens, Francelaville.solene@chu-amiens.fr (S.M.L.); 4 MP3CV Laboratory, Jules Verne University of Picardie, F-80054 Amiens, France; 5 Service de Néphrologie, CHRU de Nancy, F-54000 Vandoeuvre-lès-Nancy, France; l.frimat@chru-nancy.fr; 6 Université de Lorraine, APEMAC, F-54000 Nancy, France; 7 Service de Néphrologie Transplantation Dialyse Aphérèse, Centre Hospitalier Universitaire de Bordeaux, F-33076 Bordeaux, France; christian.combe@chu-bordeaux.fr; 8 Inserm U1026, Université Bordeaux Segalen, F-33076 Bordeaux, France; 9 Service de Néphrologie, Centre Hospitalier Lyon Sud, Université de Lyon, Carmen, F-69495 Pierre-Bénite, France; denis.fouque@univ-lyon1.fr; 10 Université Claude Bernard Lyon 1, Carmen INSERM U1060, F-69495 Pierre-Bénite, France; 11 Agence de la Biomédecine, F-93212 Saint-Denis La Plaine, France; 12 Bordeaux Population Health Center, INSERM U1219, 146 rue Léo Saignat, F-33076 Bordeaux, France; catherine.helmer@u-bordeaux.fr; 13 Association Pour L’Utilisation du Rein Artificiel dans la Région Parisienne (AURA), 185a rue Raymond Losserand, F-75014 Paris, France; 14 Ambroise Paré University Hospital, APHP, Department of Nephrology, 9 Avenue Charles de Gaulle, F-92100 Boulogne-Billancourt, France

**Keywords:** chronic kidney disease, urea, depression, kidney brain axis

## Abstract

Depression is common in patients with chronic kidney disease (CKD). Experimental studies suggest the role of urea toxicity in depression. We assessed both the incidence of antidepressant prescriptions and depressive symptoms (measured by CESD (Center for Epidemiologic Depression) scale) in 2505 patients with CKD (Stage 3–4) followed up over 5 years in the Chronic Kidney Disease Renal Epidemiology and Information Network (CKD-REIN) cohort. We used a joint model to assess the association between the serum urea level and incident antidepressant prescriptions, and mixed models for the association between the baseline serum urea level and CESD score over the 5-year follow-up. Among the 2505 patients, 2331 were not taking antidepressants at baseline. Of the latter, 87 started taking one during a median follow-up of 4.6 years. After adjustment for confounding factors, the hazard ratio for incident antidepressant prescription associated with the serum urea level (1.28 [95%CI, 0.94,1.73] per 5 mmol/L increment) was not significant. After adjustment, the serum urea level was associated with the mean change in the CESD score (β = 0.26, [95%CI, 0.11,0.41] per 5 mmol/L increment). Depressive symptoms burden was associated with serum urea level unlike depression events. Further studies are needed to draw firm conclusions and better understand the mechanisms of depression in CKD.

## 1. Introduction

While the lifetime prevalence of depression in the general population ranges from 7 to 12% in men and from 20 to 25% in women, it is higher in patients with chronic kidney disease (CKD); values from 26% to almost 40% have been reported, depending on the CKD stage and the depression scale used [1,2]. Depression is one of the most prominent psychiatric disorders in patients with CKD. The significant symptom burden can affect quality of life, adherence to care, and decision-making. Furthermore, the literature data show that depressive symptoms are associated with a higher mortality rate among patients with CKD [3,4]. Consequently, the management of depression and the identification of risk factors are of crucial importance for patients with CKD.

Elevated urea levels are particularly common in patients with moderate-to-severe CKD. As the end-product of protein metabolism, urea has a vital role in the urine-concentrating process and water conservation and is also a marker of the severity of kidney disease and the requirement for dialysis. Acute, high serum urea concentrations are not only associated with uremic syndrome but also have direct and indirect chronic toxic effects [5,6]. Although the mechanisms underlying urea toxicity have not been fully characterized, recent studies have highlighted the impact of high serum urea levels on cardiovascular events and death rates [7,8]. On one hand, urea’s direct toxicity appears to be exerted through the activation of pro-inflammatory proteins and elevated oxidative stress, which leads to an increase in pro-apoptotic gene expression, free radical levels, and senescence. On the other hand, urea’s indirect toxicity is exerted through carbamylation processes, i.e., the non-enzymatic, post-translational modification of proteins via a reaction with cyanate (a subproduct of urea) [9,10]. Carbamylation alters protein structures and functions and is associated with atherosclerosis, lipid metabolism, immune system dysfunction, renal fibrosis, and other types of damage [11,12,13,14].

Depression is associated with multiple factors, such as stress, genetic predispositions, and traumatic life events. The results of recent studies suggest that chronic inflammation has a key role in the induction of neuroinflammation and depression [15,16,17], as has already been shown for many other chronic diseases (such as cardiovascular disease, atherosclerosis, CKD, and cancer) and in the physiology of cellular aging and immunosenescence [18,19,20]. Similarly, inflammatory and oxidative stress markers are significantly and positively associated with the presence of depression [21,22,23,24]. Moreover, recent research has suggested a link between depression and carbamylation-induced impairments in synaptic plasticity [25].

Although urea exerts toxicity through several pathways (notably by promoting a pro-inflammatory state), it is not known whether urea accumulation is an independent risk factor for depression. The objective of the present study was to evaluate the putative association between the serum urea level and depression in a large cohort of non-transplanted, non-dialysis-dependent patients with CKD. We hypothesized that higher serum urea levels are associated with incident depression or higher burden of depressive symptoms in CKD population.

## 2. Results

### 2.1. Baseline Characteristics of the Study Population

**Figure 1:**
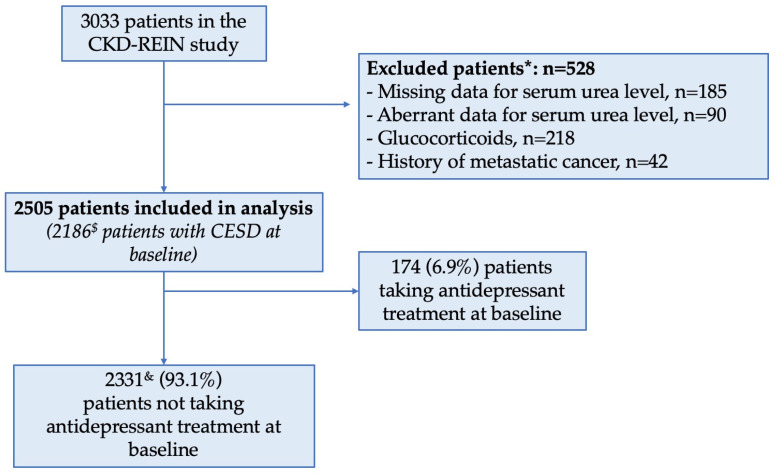
Study flowchart. ^&^ Patients included in the analysis on the initiation of antidepressant treatment. ^$^ Patients included in the analysis on the burden of depressive symptoms. * Patients may have been excluded for several reasons.

Of the 2505 patients included in all or some of the analyses, 174 (6.9%) were taking antidepressants at baseline (Figure 1). The mean (standard deviation (SD)) age of patients was 67 (13), and 34% were female. The mean (SD) eGFR was 33.4 (12.8) mL/min/1.73 m^2^ (Table 1), and the mean (SD) baseline serum urea concentration was 14.3 (6.4) mmol/L. Patients with higher serum urea concentrations at baseline were older (*p* = 0.01) and more likely to have poor cognitive performance (*p* < 0.001), depressive symptoms (*p* = 0.002), cardiovascular disease (*p* < 0.001), and higher CRP concentrations (*p* < 0.001) (Table 1).

### 2.2. Association between Incident Depression and the Serum Urea Level during the 5-Year Follow-Up Period

**Figure 2:**
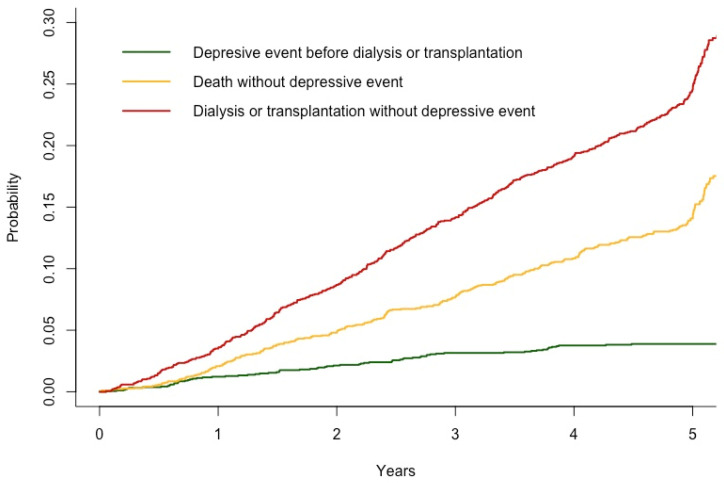
Cumulative incidence of a depressive event, death without a depressive event, and dialysis or transplantation without a depressive event (n = 2331).

Among the 2331 patients who were not taking an antidepressant at baseline, 87 initiated antidepressant treatment during the 5 years of follow-up. The mean follow-up was 3.9 years (SD, 1.5). Figure 2 represents the estimated cumulative incidence of a depression event and the competing risks (death and KRT).

**Figure 3:**
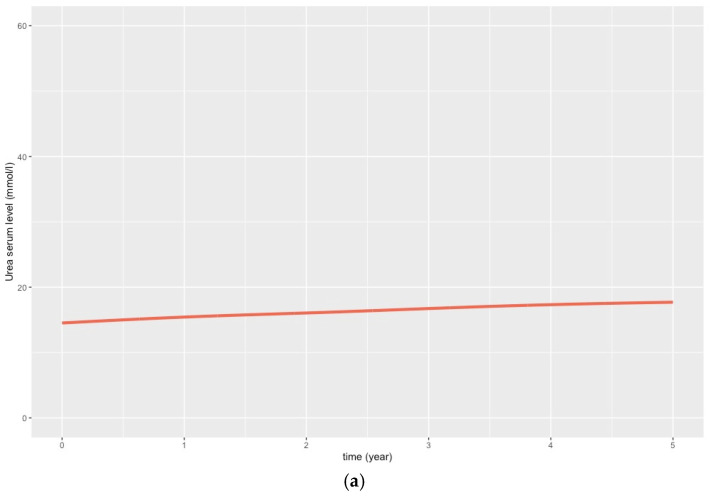

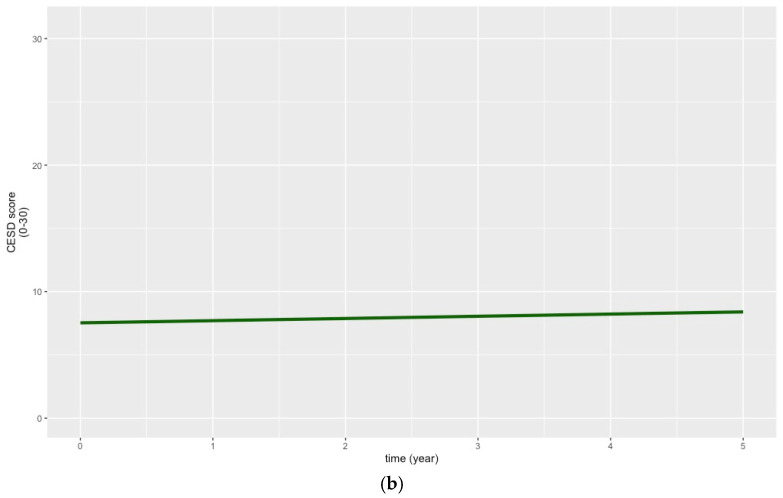
Predicted mean trajectories for the serum urea concentration and the mean CESD (Center for Epidemiologic Depression) score over the 5-year follow-up period. (**a**) Predicted mean trajectory for the serum urea concentration over the 5-year follow-up period, using a three-node cubic spline mixed model. Urea serum level was measured as part of routine CKD care. (**b**) Predicted linear trajectory for the mean CESD score over the 5-year follow-up period. The CESD scale was scored at baseline and at the 1-, 2-, 3- and 5-year follow-up visits.

For our main analysis, we predicted the trajectories of the serum urea concentration and the eGFR over the 5-year follow-up period for 1882 patients, i.e., those with full data for all the variables included in the models (Figure 3a).

In the unadjusted joint model (Model 1), a depression event (the initiation of antidepressant treatment) was not associated with either the serum urea value (hazard ratio (HR) [95% confidence interval (CI)]: 0.99 [0.76 to 1.27] for a 5 mmol/L increment; *p* = 0.9) or the slope (HR [95%CI]: 1.55 [0.84 to 2.83] for a 5 mmol/L increment; *p* = 0.2) (Table 2). After adjustment (Model 2) or after taking account of the eGFR (Model 3), the serum urea level was still not associated with a depression event. Similar results were obtained in the two sensitivity analyses (one after the imputation of missing data and the other of the highest urea tertile (>15.52 mmol/L)).

### 2.3. Association between the Baseline Serum Urea Level and the CESD Score over the 5-Year Follow-Up

The CESD score was measured at baseline for 2186 patients (Figure 1); the mean (SD) value was 7.5 (5.2). During the follow-up period, more than half of these patients completed the CESD at least four times. The mean (SD) time interval between the baseline measurement and the last measurement during follow-up was 3.8 (1.6) years. Figure 3b shows the predicted linear trajectory of the CESD over the 5 years of follow up, according to the unadjusted linear mixed model (Model 1).

In the unadjusted linear mixed model (Model 1), a higher serum urea level was significantly associated with a higher mean CESD (β [95%CI]: 0.36 [0.21 to 0.52] per 5 mmol/L increment; *p* < 0.001) (Table 3). In Model 2, we observed an association between the serum urea level and the mean change in the CESD score (β [95%CI]: 0.26 [0.11 to 0.41] per 5 mmol/L increment; *p* < 0.001). In Model 3, we observed a significant interaction between the serum urea level and the eGFR (*p* = 0.004); this led to a stronger association between the serum urea level and mean CESD for higher eGFR values (Table 3). We did not observe an interaction between the serum urea level and the serum CRP level (Model 2, *p* = 0.8).

## 3. Discussion

In this longitudinal study of a large CKD population with 5 years of follow up, we did not observe a statistically significant association between the serum urea level and the onset of an incident depression event. However, we did observe a significant association between the serum urea level and the depressive symptom burden during the 5-year follow-up period.

To the best of our knowledge, this is the first longitudinal epidemiological study to assess the association between serum urea and depression in a large cohort of patients with CKD but who were not on KRT. We assessed depression in two different ways: as the initiation of antidepressant treatment in the main analysis and as the depressive symptom burden (according to a short form of the CESD scale) in the secondary analysis. The antidepressants studied were those prescribed specifically for depression (ATC codes N06AB and N06AX), and the prescriptions were re-evaluated annually. In the absence of a validated cut-off for depression in the short-form CESD (in contrast to the full scale), we decided not to dichotomize the score. The short-form CESD scale is more appropriate as a screening tool for depressive symptoms, rather than depression per se. We adjusted the models for a history of depression, since previously depressed patients were more likely to present a new episode of depression.

In a recent study of a mouse model of CKD and patients in the uremic phase of CKD, Wang et al. found that urea accumulation induced depression directly (i.e., without the requirement for psychosocial stress) [25]. Furthermore, Wang et al.’s findings suggested that the underlying mechanism involved carbamylated for mTOR, inhibition of mTORC1-S6K-dependent dendritic protein synthesis, impaired synaptic plasticity in the prefrontal cortex, and thus depression-like behavior. However, Wang et al.’s study included only 40 patients, all of whom were on hemodialysis; hence, the long-term uremic load was probably high. The mean serum urea concentration reported by Wang et al. was above 20 mmol/L, and the variations between pre- and post-dialysis measurements were probably significant. In our study, the mean serum urea level was 14 mmol/L, and the patients’ values were relatively stable throughout the 5 years of follow-up. Hence, we expected that the uremic load was lower among our patients than among Wang et al.’s patients. Furthermore, it was reported recently that urea has cardiovascular toxicity at levels above 15 mmol/L [7].

In contrast to Wang et al.’s models, our models were adjusted for a large number of confounding factors. Even though the association between the serum urea value and the onset of a depression event was not statistically significant, the corresponding HR had a confidence interval close to one. The value of the urea level appeared to have more influence on the occurrence of depression events than the slope did.

Our secondary analysis of the association between the baseline serum urea level and the depressive symptom burden during follow up revealed a significant interaction between the serum urea level and the eGFR; this offered a stronger association between the serum urea level and the mean CESD score for higher eGFR values. We believe that in patients with a higher eGFR, a high urea level is rare or reflects an additional acute impairment (major volume variations, an iatrogenic factor, etc.). These unusually high serum urea concentrations have a greater impact on patients’ uremic symptoms in general and on depressive symptoms in particular.

In our study, the serum urea level was more strongly associated with depressive symptoms than it was with depressive disease. Despite the absence of a significant association between serum urea and depression events in our cohort, we believe that further studies are needed because (i) the 95%CI for the HR provided by our statistical models was very close to one and (ii) the serum urea level was associated with depressive symptom burden. Indeed, several recent fundamental studies have generated robust physiological evidence on an impact of urea and cyanate (via carbamylation processes) on oxidative stress, apoptosis, and synaptic plasticity [13,14,25]. Moreover, high serum urea levels are associated with accelerated atherosclerosis [7]. A study in an animal model showed that urea transporter B knock-out mice (in which urea accumulated in the blood and brain) displayed depression-like behavior [26]. Studies in various patient populations (hemodialyzed vs. not, older vs. younger, and with different comorbidity burdens) might enable researchers to determine whether or not there is a serum urea or uremic load threshold beyond which neurotoxicity appears. Although levels of carbamylation are particularly significant in patients with CKD (due to the high serum urea concentration), this process also occurs throughout life; indeed, some experts consider carbamylation to be a hallmark of aging [27]. Thus, the elevated serum urea concentration seen in CKD might accelerate aging in general and cerebral aging and the associated pathologies (particularly depression and neurocognitive disorders). Studies of urea and uremic toxins in CKD might lead to a better understanding of the components of the kidney–brain axis.

Our study has several strengths. Firstly, a large number of patients (more than 2000) were included. Secondly, many potential confounding factors were taken into account. Thirdly, we assessed depression in terms of both treated illness (through the prescription of antidepressants) and the symptom burden (using a shirt-form CESD scale). Fourthly, the CESD score was rated regularly during the follow-up period, and the mixed-model design allowed us to take account of all the patients with CESD data and not just those to whom the CESD questionnaire was administered several times during follow-up. Lastly, the use of joint models enabled us to take account of urea values over the entire follow-up period and to adjust the data for the eGFR during the same period; this enabled us to look for a specific effect of the serum urea level, independently of the CKD stage. 

However, our study had a number of limitations. Firstly, it was subject to the selection bias inherent in all cohort studies because depressed patients are often less motivated to undergo follow-up procedures and to answer self-questionnaires. These selection and classification biases might have affected our results concerning the association between the serum urea level and depression by (i) excluding the more depressed patients from inclusion in the cohort and (ii) reducing the number of depression events reported and the number of depressed patients completing the CESD questionnaire. Secondly, the baseline prevalence of depression in our cohort (assessed as being treated with an antidepressant) was 6.9%; this value is lower than those reported for other studies of CKD patients, which range from 26% to 40% (depending on the CKD stages and the depression scale used). This relatively low prevalence could be interpreted by symptoms less frequently reported by our patients and therefore less considered by physicians [1,2,28,29]. Thirdly, the patients with higher serum urea level or a rapid increase in that level were often the most severely ill and were more likely to experience dialysis, initiation, kidney transplantation, or death. As a result, these patients are likely to have been followed up for shorter periods. Hence, data on depression was more likely to be lost because in our study, the active follow-up stopped upon initiation of KRT. Fourthly, the CESD scale was the only scale used to assess the depressive symptom burden in our cohort. In contrast to the full CESD scale, a cut-off score for depression was not validated in the short-form CESD [30]. We were therefore unable to use the CESD score as a criterion for depression (as a disease) in our main analysis. Lastly, and even though we rated the short-form CESD score several times over the 5-year follow-up period, convergence problems prevented us from using a random slope in our mixed models.

## 4. Conclusions

The present longitudinal study was the first to evaluate the association between the serum urea level and depression in patients with CKD but not having started KRT. We did not observe a statistically significant association between the serum urea level and depression events, whereas we did observe a significant association between the serum urea level and depressive symptoms, depending on the eGFR. In view of the recent research highlighting the toxicity of urea, we believe that further studies are needed to draw firms conclusions and gain a better understanding of the mechanisms underlying depression in CKD.

## 5. Materials and Methods

### 5.1. Participants

We analyzed data from the Chronic Kidney Disease-Renal Epidemiology and Information Network (CKD-REIN) cohort (ClinicalTrials.gov identifier: NCT 03381950) [31]. This cohort comprises 3033 CKD Stage 3 or 4 patients recruited at 40 randomly selected nephrology facilities in mainland France between July 2013 and April 2016 [31]. Patients were eligible if they had an eGFR below 60 mL/min/1.73 m^2^ (measured twice, at least one month apart) and were not receiving kidney replacement therapy (KRT). Furthermore, the patients had to be aged 18 or over and able to provide written informed consent. The study design involved a routine visit every year for the 5 years of follow-up or up to 6 months after the initiation of KRT. The cohort protocol was approved by the institutional review board at the French National Institute of Health and Medical Research (Institut National de la Santé et de la Recherche Médicale, Paris, France; reference: IRB 00003888).

For the purposes of the present analysis, we excluded patients with missing data on the serum urea level at baseline or an aberrant baseline value (<2.5 or >100 mmol/L). To limit the effects of non-renal factors on serum urea levels, patients who were on corticosteroids or had a history of metastatic cancer were also excluded (Figure 1).

### 5.2. Data

Baseline and follow-up data were collected by clinical research associates from medical records, patient interviews, and patient self-questionnaires.

The serum urea level was expressed in mmol/L. The eGFR was estimated using the 2009 creatinine-based Chronic Kidney Disease Epidemiology Collaboration equation with the ethnic variable (CKD EPI) [32]. All patients were prescribed a set of standard blood and urine tests (recommended by French health authorities as part of routine CKD care) to be performed at their usual medical laboratory.

The study data included baseline sociodemographic variables (age, sex, educational level, living alone or not, personal independence (assessed on an activities of daily living scale and an instrumental ADL (IADL) scale), physical activity (based on the Global Physical Activity questionnaire score and professional status); overall cognitive performance (assessed in the Mini Mental State Examination (MMSE); medications (notably polymedication and the intake of antidepressant and anxiolytic drugs); cardiovascular risk factors and comorbidities (hypertension, diabetes mellitus, dyslipidemia, smoking status, cerebrovascular disease, and cardiovascular disease); and laboratory data (notably the urine albumin-to-creatinine ratio and the serum level of C-reactive protein (CRP)). Further details of these variables are given in Supplementary Table S1 [33,34,35,36].

### 5.3. Measurement of Depression

Firstly, we assessed the incidence of a depression event; the latter was defined as the initiation of treatment with an antidepressant (Anatomical Therapeutic Chemical (ATC) codes N06AB and N06AX) during the 5-year follow-up period. In our main analysis, patients with depression at baseline (defined as the prescription of an antidepressant in the 3 months prior to inclusion in the cohort) were excluded. At each annual follow-up visit, patients were asked to bring all their prescriptions from the previous year. We recorded the ATC class, the unit dose, and the prescription start and end dates. When the date of prescription was not known, it was imputed as the date midway between the previous follow-up visit and the follow-up visit at which the prescription status was noted.

Secondly, we assessed the change over time in depressive symptoms by applying the Center for Epidemiologic Studies Depression (CESD) scale [37,38]. The original CESD scale comprises 20 items. Each item is scored from zero (“Rarely or never”, i.e., less than one day in the past week) to three (“Most of the time”, i.e., 5–7 days a week). In the CKD-REIN cohort, a short (10-item) self-questionnaire version of the CESD (i.e., with a score out of 30) was used [39]. The CESD scale was scored at baseline and at the 1-, 2-, 3- and 5-year follow-up visits.

### 5.4. Statistical Analysis

We described the patients’ baseline characteristics as a function of the baseline serum urea concentration in tertiles: <10.8 mmol/L, between 10.8 and 15.51 mmol/L, and >15.52 mmol/L (Table 1). An analysis of variance was applied to continuous variables, and a chi-squared test was applied to categorical variables. 

In the main analysis, we first estimated the cumulative incidence of a depression event and the competing risks (death or KRT, using cuminc function from cmprsk package) and checked the proportional hazard assumption [40]. Next, we used a joint model approach to estimate the associations between the current value and slope of the serum urea variable and the initiation of antidepressant treatment [41]. Patients were followed up until death, KRT initiation, loss to follow up, or completion of the 5-year follow up, whichever occurred first. The joint model is based on a mixed model (for longitudinal variables) and a cause-specific proportional hazard model. We first developed a “crude” joint model (including only the serum urea value and slope; Model 1) and then a model adjusted for baseline confounding factors identified in a directed acyclic graph: age, sex, MMSE score, hypertension, diabetes mellitus, dyslipidemia, body mass index, current smoking, and serum CRP level (Model 2) [42]. To assess the specific effect of urea on the occurrence of a depression event, we adjusted the multivariate model for the eGFR value and slope (per 10 mL/min increment) over the 5 years of follow-up (Model 3). In order to estimate individual urea and eGFR trajectories with more accuracy and flexibility, restricted cubic splines with four degrees of freedom (three knots) were applied to the follow-up time in the linear mixed-effects part of the model. The Bayesian approach to joint modelling (using the JMbayes2 package in R software (2023.06.2+561 version) [43]) does not require random effects to be normally distributed. The homoscedasticity of the mixed-effects part’s residuals was checked graphically. Convergence of the Markov chain Monte Carlo algorithm (used to estimate the coefficients of the joint model) was evaluated graphically with a maximum of 50,000 iterations. Patients with missing data were excluded from this analysis. In an initial sensitivity analysis of the joint models, we performed simple imputation using the nonparametric missForest algorithm [44]. All the variables used in the joint models and mixed models were included in the imputation algorithm. For each missing variable, a random forest with 100 trees was built. In view of our hypothesis whereby a higher serum urea level would lead to more incident depression events, we performed a second sensitivity analysis on patients in the upper tertile of baseline serum urea levels.

Secondly, we used linear mixed-effects models with a random intercept to analyze the association between the baseline serum urea level and the changes over time in CESD scores in patients with at least one CESD score at baseline. We used the same adjustment factors as in the joint model described above. A random slope could not be introduced into the model because of convergence concerns. To assess the association between the baseline serum urea level and changes over time in the CESD score, we tested the interaction term between the baseline serum urea level and time. With regard to a putative microinflammatory mechanism, we also included the interactions between serum urea and eGFR and between serum urea and serum CRP in the model. The homoscedasticity of the mixed model residuals was checked graphically. Linear mixed models were built with the lmer function in the lme4 package in R [45]. We used chained equations to perform multiple imputations of missing data [46]. A total of 30 datasets were created with 30 iterations. All variables present in the linear mixed model were included in the imputation procedure.

All statistical analyses were performed with R software (version 4.1.3) [43].

## Figures and Tables

**Table 1:** Baseline characteristics of the participants (n = 2505), according to the serum urea level at baseline.

	NA n (%)	Overall n = 2505	1st Tertile <10.8 mmol/L n = 835	2nd Tertile 10.8–15.51 mmol/L n = 835	3rd Tertile ≥15.52 mmol/L n = 835	*p* *
Age, mean (SD)	0	67.2 (12.7)	66.44 (12.1)	67.39 (13.2)	67.74 (12.6)	**0.01**
Female sex, %	0	853 (34.1)	281 (33.7)	285 (34.1)	287 (34.4)	0.9
Antidepressants, %	0	174 (6.9)	58 (6.9)	65 (7.8)	51 (6.1)	0.4
Anxiolytics, %	0	263 (10.5)	99 (11.9)	85 (10.2)	79 (9.5)	0.3
Polymedication (≥5 drugs/d), %	0	2012 (80.3)	586 (70.2)	690 (82.6)	736 (88.1)	**<0.001**
CESD-10 (0–30), mean (SD)	319 (12.7)	7.5 (5.2)	6.96 (4.9)	7.64 (5.4)	7.85 (5.2)	**0.002**
History of depression, %	0 (0)	189 (7.5)	77 (9.2)	60 (7.2)	52 (6.2)	0.2
Living alone, %	365 (14.6)	496 (19.8)	150 (18.0)	162 (19.4)	184 (22.0)	0.3
ADL (out of 5), mean (SD)	287 (11.5)	4.9 (0.4)	4.93 (0.4)	4.92 (0.4)	4.90 (0.4)	0.5
IADL (out of 8), mean (SD)	294 (11.7)	7.2 (1.3)	7.43 (1.0)	7.23 (1.3)	7.01 (1.5)	**<0.001**
Sedentary (GPAQ, class 3), %	412 (16.4)	995 (39.7)	304 (36.4)	323 (38.7)	368 (44.1)	**0.006**
Educational level ≥ 12 years, %	33 (1.3)	884 (35.3)	338 (40.5)	294 (35.2)	252 (30.2)	**0.001**
MMSE score (out of 30), mean (SD)	23 (0.9)	26.9 (3.0)	27.24 (2.8)	26.81 (3.1)	26.55 (3.0)	**<0.001**
Professional status, %	311 (12.4)					
Employed		375 (15.0)	141 (16.9)	124 (14.9)	110 (13.2)	0.3
Retired		1618 (64.6)	528 (63.2)	540 (64.7)	550 (65.9)	
Unemployed		201 (8.0)	58 (6.9)	68 (8.1)	75 (9.0)	
***Cardiovascular risk factors*** ^ϒ^						
Hypertension, %	3 (0.1)	2296 (91.7)	736 (88.1)	776 (92.9)	784 (93.9)	**<0.001**
Diabetes mellitus, %	6 (0.2)	1111 (44.4)	306 (36.6)	367 (44.0)	438 (52.5)	**<0.001**
Dyslipidemia, %	4 (0.2)	1867 (74.5)	576 (69.0)	633 (75.8)	658 (78.8)	**<0.001**
Obesity, %	48 (1.9)	889 (35.5)	243 (29.1)	295 (35.3)	351 (42.0)	**<0.001**
BMI, mean (SD)	48 (1.9)	28.9 (5.8)	28.24 (5.2)	28.80 (5.9)	29.56 (6.3)	**<0.001**
Current smoking, %	20 (0.8)	313 (12.5)	104 (12.5)	93 (11.1)	116 (13.9)	0.2
***Comorbidities***						
Cardiovascular disease ^θ^, %	32 (1.3)	1344 (53.7)	400 (47.9)	450 (53.9)	494 (59.2)	**<0.001**
Cerebrovascular disease ^¥^, %	62 (2.5)	306 (12.2)	86 (10.3)	101 (12.1)	119 (14.3)	0.2
Heart failure ^Ψ^, %	6 (0.2)	325 (13.0)	71 (8.5)	100 (12.0)	154 (18.4)	**<0.001**
***Laboratory variables***						
eGFR (mL/min/1.73 m^2^), mean (SD)	0	33.4 (12.8)	44.39 (10.9)	32.52 (9.6)	23.41 (7.8)	**<0.001**
CRP serum level (mg/L), mean (SD)	405 (16.2)	3.8 (3.9)	3.37 (3.6)	3.73 (3.8)	4.22 (4.2)	**<0.001**
Urinary albumin/creatinine ratio, %	201 (8.0)					
<3 mg/mmol		618 (24.7)	308 (36.9)	196 (23.5)	114 (13.7)	**<0.001**
3 to 30 mg/mmol		720 (28.7)	246 (29.5)	255 (30.5)	219 (26.2)	
>30 mg/mmol		966 (38.6)	213 (25.5)	312 (37.4)	441 (52.8)	

^ϒ^: Cardiovascular risk factors: Hypertension was defined as a history of hypertension or the use of blood-pressure-lowering medication. Diabetes mellitus was defined as a history of diabetes, antidiabetic medication use, a glycosylated hemoglobin level ≥ 6.5%, a fasting glycemia value ≥ 7 mmol/L, or a non-fasting glycemia value ≥ 11 mmol/L. Dyslipidemia was defined as a history of dyslipidemia or the use of lipid-lowering medication. Obesity was defined as a body mass index ≥ 30 kg/m^2^. Current smoking was defined as at least one cigarette per day. ^θ^: History of coronary heart disease, angina pectoris, myocardial infarction, coronary bypass surgery, percutaneous coronary intervention, cardiac arrest, atrial fibrillation, other heart rhythm disorder, an implanted pacemaker, implanted defibrillator, heart failure, pulmonary edema, pericarditis, heart valve disease, heart valve prosthesis, stroke, transient ischemic attack, endarterectomy, intracerebral hemorrhage, peripheral vascular disease, intermittent claudication, arterial bypass/percutaneous intervention for arteritis, renal artery stenosis/renal artery surgery, aortic aneurysm, and/or surgical treatment of an aortic aneurysm. ^¥^: Cerebrovascular disease was defined as a history of stroke, transient ischemic attack, or cerebral hemorrhage. ^Ψ^: History of heart failure or pulmonary oedema. * Analysis of variance for continuous variables and a chi-squared test for categorical variables. Abbreviations: NA, not available; SD, standard deviation; MMSE, Mini Mental State Examination; eGFR, estimated glomerular filtration rate; CESD-10, Center for Epidemiologic Studies Depression, 10 items; ADL, activities of daily living; IADL, instrumental activities of daily living; GPAQ, Global Physical Activity Questionnaire; BMI, Body mass index; CRP, C-reactive protein.

**Table 2:** The association between the serum urea level with depressive events over the 5-year follow-up, in unadjusted and adjusted joint models.

	Model 1		Model 2		Model 3	
	HR [95%CI]	*p*	HR [95%CI]	*p*	HR [95%CI]	*p*
Serum urea level (per 5 mmol/L increment)						
Value (urea)	0.99 [0.76, 1.27]	0.9	0.92 [0.73, 1.15]	0.5	1.28 [0.94, 1.73]	0.1
Slope (urea)	1.55 [0.84, 2.83]	0.2	1.68 [0.93, 2.94]	0.08	1.70 [0.61, 4.76]	0.3

Unadjusted and adjusted joint model (number of patients = 1882; number of events = 72). Model 1 was built from an unadjusted mixed model for longitudinal serum urea values and an unadjusted Cox model for depressive events. Model 2 was built from an adjusted mixed model for the longitudinal serum urea level values (per 5 mmol/L increment) and an adjusted Cox model for depressive events. Model 3 was built from an adjusted mixed model for the longitudinal serum urea level values (per 5 mmol/L increment), an unadjusted mixed model for the longitudinal estimated glomerular filtration rate values, and an adjusted Cox model for depressive events. Adjustments mention above were for baseline values for age, sex, Mini Mental State Examination (MMSE) score, history of depression at baseline, hypertension, diabetes mellitus, dyslipidemia, current smoking, Body Mass Index (BMI), and serum C-reactive protein. Abbreviations: HR, hazard ratio; CI, confidence interval.

**Table 3:** Associations between baseline risk factors and the mean CESD during the follow-up period.

	Mean CESD Score Model 1 ^$^		Mean CESD Score Model 2 ^£^		Mean CESD Score Model 3 ^θ^	
	Crude Effect [95%CI]	*p*	Adjusted Effect [95%CI]	*p*	Adjusted Effect [95%CI]	*p*
Time (years)	0.17 [0.12, 0.21]	**<0.001**	0.18 [0.13, 0.22]	**<0.001**	0.18 [0.13, 0.22]	**<0.001**
Urea serum level (per 5 mmol/L increment)	0.36 [0.21, 0.52]	**<0.001**	0.26 [0.11, 0.41]	**<0.001**		
Urea serum level (per 5 mmol/L increment) × eGFR						0.04
eGFR = 45 mL/min/1.73 m^2^					0.44 [0.43, 0.45]	
eGFR = 30 mL/min/1.73 m^2^					0.23 [0.22, 0.24]	
eGFR = 15 mL/min/1.73 m^2^					0.02 [0.007, 0.03]	
Serum urea level × time	0.04 [−0.006, 0.08]	0.09	0.04 [−0.007, 0.08]	0.1	0.03 [−0.008, 0.08]	0.1
Serum urea level × CRP level			−0.005 [−0.04, 0.03]	0.8	−0.003 [−0.04, 0.03]	0.9

The follow-up period was considered as a continuous variable. Unadjusted and adjusted linear mixed regressions (n = 2186). ^$^ Model 1 was an unadjusted mixed model. ^£^ Model 2 was adjusted for age, sex, history of depression, Mini Mental State Examination score, hypertension, diabetes mellitus, dyslipidemia, body mass index, current smoking, and serum CRP level. ^θ^ Model 3 was adjusted for age, sex, history of depression, Mini Mental State Examination score, hypertension, diabetes mellitus, dyslipidemia, body mass index, current smoking, serum C-reactive protein, and eGFR. All models were adjusted for the time interval between the first (baseline) CESD score and the latest CESD score. Multiple imputation by chained equations was applied to the dataset. Abbreviations: CESD, Center for Epidemiologic Studies Depression; CI, Confidence interval; eGFR, Estimated glomerular filtration rate; CRP, C-reactive protein.

## Data Availability

The data that support the findings of this study are available upon reasonable request by contacting the CKD-REIN study coordination staff at ckdrein@inserm.fr.

## Supplementary Materials

The following supporting information can be downloaded at: https://www.mdpi.com/article/10.3390/toxins16070326/s1, Table S1: Definitions of the operational variables used in the study (PDF).
