# Supplementary material for: Urea Level and Depression in Patients with Chronic Kidney Disease
Source: Toxins (Basel). 2024 Jul 22;16(7):326. doi: 10.3390/toxins16070326 (PMC11281192; doi:10.3390/toxins16070326)
Supplement: Supplementary file 1 [file toxins-16-00326-s001.zip › toxins-3077918-supplementary.pdf]

SUPPLEMENTARY MATERIAL

Supplementary Table S1: Definitions of the operational variables used in the study.

Variables	Definitions
Educational level	Educational level was assessed as the number of years in full-time education; in France, a level of 12 years or more corresponds to high school graduation.
MMSE score[36]	The Mini Mental State Examination is a 30-item questionnaire assessing global cognitive function and is frequently used to screen for neurocognitive disorder.
Activities of daily living (ADL) scale[33]	The ADL scale contained five items: eating, bathing, dressing, transferring, and toileting.
The instrumental activities of daily living (IADL) scale[34]	The IADL scale measured were telephone use, transportation use, shopping, meal preparation, housekeeping, laundry, medication handling, and money management.
Sedentarity - The Global Physical Activity Questionnaire (GPAQ)[35]	The Global Physical Activity Questionnaire (GPAQ) collects information about physical activity in work, travel, and entertainment. Based on the final score, patients were categorized as sedentary, moderately physically active, or very physically active and then dichotomized as sedentary or active.
Professional status	The professional status was categorized as employed, retired or unemployed.
Polymedication	Polymedication was defined as taking at least 5 prescription drugs per day. A specific case report form was used to record drug prescriptions during the three months prior to study inclusion.
Anxiolytics	Anxiolytics were defined as drugs in ATC class N05B
Hypertension	Hypertension was defined as a history of hypertension or the use of blood-pressure-lowering medication.
Diabetes mellitus	Diabetes mellitus was defined as a history of diabetes, antidiabetic medication use, a glycosylated hemoglobin level ≥6.5%, a fasting glycemia value ≥7 mmol/L, or a non-fasting glycemia value ≥11 mmol/L.
Dyslipidemia	Dyslipidemia was defined as a history of dyslipidemia or the use of lipid-lowering medication.
Obesity	Obesity was defined as a body mass index ≥30 kg/m^2.
Current smoking	Current smoking was noted when patients smoked at least one cigarette per day.
Cerebrovascular disease	Cerebrovascular disease was defined as a history of stroke, carotid endarterectomy, or cerebral hemorrhage.
The urine albumin-to-creatinine ratio (uACR)	The uACR was classified as normal (<3 mg/mmol), moderately high (3 to 30 mg/mmol), or very high (≥30 mg/mmol).
Laboratory data	C-reactive protein (mg/L)
